# Deletion of the ASFV dUTPase Gene *E165R* from the Genome of Highly Virulent African Swine Fever Virus Georgia 2010 Does Not Affect Virus Replication or Virulence in Domestic Pigs

**DOI:** 10.3390/v14071409

**Published:** 2022-06-28

**Authors:** Elizabeth A. Vuono, Elizabeth Ramirez-Medina, Sarah Pruitt, Ayushi Rai, Nallely Espinoza, Ediane Silva, Lauro Velazquez-Salinas, Douglas P. Gladue, Manuel V. Borca

**Affiliations:** 1 Plum Island Animal Disease Center, Agricultural Research Service, United States Department of Agriculture, Greenport, NY 11944, USA; elizabeth.vuono@usda.gov (E.A.V.); elizabeth.ramirez@usda.gov (E.R.-M.); sarah.pruitt@usda.gov (S.P.); ayushi.rai@usda.gov (A.R.); nallely.espinoza@usda.gov (N.E.); ediane.silva@usda.gov (E.S.); lauro.velazquez@usda.gov (L.V.-S.); 2 Department of Pathobiology and Population Medicine, Mississippi State University, Starkville, MS 39762, USA; 3 Oak Ridge Institute for Science and Education (ORISE), Oak Ridge, TN 37830, USA

**Keywords:** ASFV (African swine fever virus), ASF (African swine fever), *E165R* gene, virus virulence, UTPase

## Abstract

African swine fever (ASF) is a frequently lethal disease of domestic and wild swine currently producing a pandemic affecting pig production in Eurasia. The causative agent, ASF virus (ASFV) is a structurally complex virus with a large genome harboring over 150 genes. One of them, *E165R*, encodes for a protein belonging to the dUTPase family. The fine structure of the purified protein has been recently analyzed and its dUTPase activity tested. In addition, it has been reported that a BA71 mutant virus, adapted to growth in Vero cells, lacking the *E165R* gene presented a drastic decreased replication in swine macrophages, its natural target cell. Herein, we report the development of a recombinant virus, ASFV-G-∆E165R, harboring the deletion of the *E165R* gene from the genome of the highly virulent field isolate ASFV Georgia 2010 (ASFV-G). Interestingly, ASFV-G-∆E165R replicates in primary swine macrophage cultures as efficiently as the parental virus ASFV-G. In addition, ASFV-G-∆E165R also replicates in experimentally inoculated domestic pigs with equal efficacy as ASFV-G and produced a lethal disease almost indistinguishable from that induced by the parental virus. Therefore, results presented here clearly demonstrated that *E165R* gene is not essential or important for ASFV replication in swine macrophages nor disease production in domestic pigs.

## 1. Introduction

African swine fever virus (ASFV) is the etiological agent of a frequently lethal disease of domestic pigs, African swine fever (ASF). Currently, ASF is widely distributed across Eurasia, affecting the swine production industry. ASF reappeared in the Dominican Republic and Haiti after more than 40 years of being absent in the Western hemisphere [1]. Since no commercial vaccine is available, and only experimental vaccines have been reported [2], ASF disease control is limited to the elimination of all infected animals and limiting the animal mobilization in the affected geographical area.

ASFV is a structurally complex virus, with a very large double-stranded DNA genome (180 to 190 Kb) encoding more than 150 genes [3]. The functions of many of these genes are still unidentified or have just been predicted by functional genomics. Understanding the function of these genes has led to the development of effective countermeasures, such as experimental, live, attenuated vaccines [4,5,6,7,8,9,10,11]. The discovery of genes involved in disease production in pigs was an essential initial step in the rational design of live, attenuated ASFV vaccine candidates [2]. Similarly, the identification of virus genes involved in critical events during the process of virus replication may constitute essential information for the potential development of therapeutic drugs to prevent or limit virus infection. Among those are virus genes, which have been studied and have been characterized as potential targets to decrease virus replication is *E165R*, which encodes for a protein with dUTPase function [12]. dUTPases are ubiquitous enzymes involved in DNA repair that play an important function in preventing uracil from being incorporated into DNA. dUTPases are widespread in organisms, including eukaryotes, prokaryotes, and some viruses [13]. The protein encoded by the *E165R* gene was purified and functionally characterized as dUTPase and, in addition, was shown to be critical for virus replication in swine macrophage cultures [12]. A recombinant virus, having deleted the *E165R*, was developed using ASFV strain BA71V, which was adapted to efficiently grow in the cell line of monkey origin, Vero, demonstrating that the absence of the *E165R* gene decreased virus replication by 90% in swine macrophages, its natural target cell. Recently, the crystal structure of the purified protein has been analyzed, the active site characterized, and its dUTPase activity confirmed [12,13,14]. In addition, monoclonal antibodies generated against the conserved areas of *E165R* have been shown to inhibit the dUTPase activity of E165R [15]. Considering the apparently essential role of *E165R* in virus replication in macrophages [12], these recent studies have raised the possibility of developing therapeutic tools to prevent or control ASFV replication based on the use of drugs or antibodies [13,14,15].

Interestingly, we report here that a recombinant virus, ASFV-G-∆E165R, having deleted the *E165R* gene in the genome of the highly virulent strain Georgia 2010 (ASFV-G), replicates in primary swine macrophage cultures as efficiently as the parental virus ASFV-G. Importantly, it is shown that ASFV-G-∆E165R also efficiently replicates in experimentally inoculated domestic pigs and produces a disease almost indistinguishable from that induced by ASFV-G. Therefore, results presented here clearly demonstrate that the ASFV *E165R* gene is not important for the processes of replication in swine macrophages nor disease production in domestic pigs.

## 2. Materials and Methods

### 2.1. Viruses and Cells

Swine macrophage cultures were produced, as previously described in detail [16], and used at a density of 5 × 10^6^ cells per ml. ASFV Georgia (ASFV-G) was a field isolate kindly provided by Dr. Nino Vepkhvadze, from the Laboratory of the Ministry of Agriculture (LMA) in Tbilisi, Republic of Georgia [17]. Growth curves between ASFV-G-∆E165R and ASFV-G were performed using primary swine macrophages in six well plates with an MOI of 0.01 HAD_50_ (hemadsorbing doses), as determined in primary swine macrophage cell cultures, as previously described [17]. Sample points were taken at 2, 24, 48, 72, and 96 h, cells were frozen at ≤−70 °C, thawed, and the lysates titrated by HAD_50_/mL in primary swine macrophage cell cultures in 96-well plates. The presence of the virus-infected cells was assessed by hemadsorption (HA) and virus titers were calculated as previously described [18].

### 2.2. Construction of the E165R-Deleted ASFV Mutant

A recombinant mutant virus harboring a deletion of the *E165R* gene (ASFV-G-∆E165R) was developed after homologous recombination of the parental virus (ASFV-G) genome and the recombination transfer vector p72mCherryΔE165R, as previously described [10]. p72mCherryΔE165R contains the genomic regions adjoining the E165R gene: the left arm located between genomic positions 166,640–167,640 and the right arm located between genomic positions 167,787–168,787 (GenBank accession number NC_044959.2). p72mCherryΔE165R also contains a reporter-gene cassette containing the mCherry fluorescent protein (mCherry) gene under the control of the ASFV p72 late-gene promoter [19]. The p72mCherryΔE165R vector was obtained by DNA synthesis (Epoch Life Sciences, Sugar Land, TX, USA). The designed construct produced a 146-nucleotide deletion between nucleotide positions 167,641–167,786, partially deleting the *E165R* ORF sequence. The recombinant ASFV-G-∆E165R was purified by consecutive limiting dilution steps based on mCherry activity detection and full-length sequenced using next-generation sequencing (NGS).

### 2.3. Next Generation Sequencing of ASFV

Virus DNA was extracted from infected macrophage cultures presenting higher than 90% CPE. The nucleus and cytoplasmic fractions were separated using a nuclear extraction kit, with the viral DNA being isolated from the cytoplasmic fraction, following the manufacturer’s protocol (Active Motif cat# 40010). ASFV infected cells were treated with the hypotonic buffer on ice until the cell membrane was dissolved. The nucleus fraction was separated by centrifugation; the cytoplasmic fraction was collected and DNA extracted adding 10% (*v*/*v*) of 3M NaAc (Sigma-Aldrich 71196, St. Louis, MO, USA) and an equal volume of phenol:chloroform:isoamyl alcohol (25:24:1) with a pH of 6.5–6.9 (Sigma-Aldrich P3803-100ML). The preparation was centrifuged on maximum speed in a tabletop centrifuge, the aqueous layer was then ethanol precipitated using 2 volumes of 100% ethanol, washed with the same volume of 70% ethanol, and dried. The resulting DNA pellet was then reconstituted in sterile water. We then used this DNA library for NGS sequencing using Nextera XT kit in the NextSeq (Illumnia, San Diego, CA, USA), following the manufacturer’s protocol. Sequence analysis was performed using CLC Genomics Workbench software (CLCBio, Waltham, MA, USA).

### 2.4. Evaluation of ASFV-G-ΔE165R Virulence in Domestic Pigs

The virulence of ASFV-G-∆E165R was assessed in 35–40 kg commercial breed swine. Five pigs were intramuscularly (IM) inoculated with 10^2^ HAD_50_ of ASFV-G-∆E165R. A similar group of animals were inoculated with 10^2^ HAD_50_ of ASFV-G. The appearance of clinical signs (anorexia, depression, fever, purple skin discoloration, staggering gait, diarrhea, and cough) and body temperature were monitored daily during the experiment. Blood samples were scheduled to be obtained at 0, 4, and 7, 11, 14, 21 and 28 days post inoculation (pi). Animal experiments were performed under biosafety level 3 conditions in the animal facilities at Plum Island Animal Disease Center, following a strict protocol approved by the Institutional Animal Care and Use Committee (number 225.06-19-R, approved 09-10-19).

## 3. Results and Discussion

### 3.1. Variability of E165R Gene across Different ASFV Isolates

To assess the genetic variability of the *E165R* gene of ASFV in nature, a total of 105 sequences were retrieved from the GenBank data base. After that, redundant sequences were removed using the software Jalview version 2.11.1.7 (Dundee, United Kingdom) obtaining a total of 9 representative field strains. These strains included: Georgia 2007/1 (genotype II), MAL/19/Karonga (genotype II), Liv13/33 (genotype I), 22653/Ca/2014 (genotype I), Pretoriuskop/96/4 (genotype XX), RSA 2 2008 (XXII), Ken05/Tk1 (genotype IX), Ken06.Bus (genotype X), and Malawi Lil-20/1 (genotype VIII). Based on a pairwise analysis conducted using the p-distance model and the bootstrap method to give statistical confidence (p-0.05) to the analysis, we are reporting an overall identity between 99.59% and 92.32% (~96.21%), and 99.39% and 91.51% (~96.89%) at nucleotide and amino acid levels, respectively.

**Figure 1:**
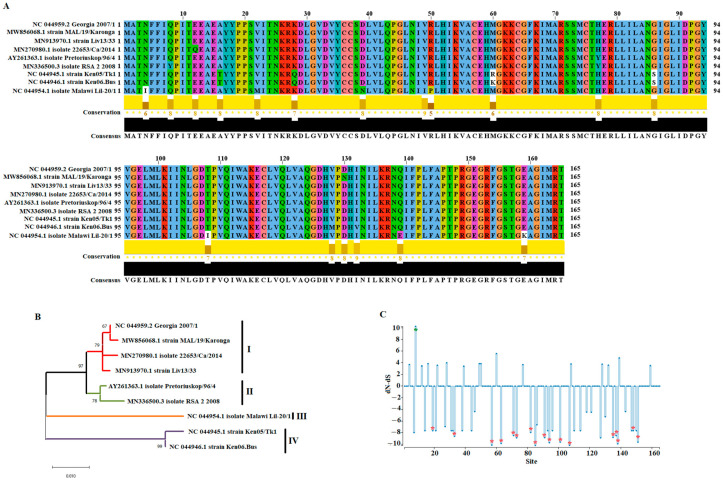
Evaluation of the *E165R* across ASFV isolates. (**A**) Amino-acid alignment representing the diversity of E165R protein of ASFV in the field. Residues in white spots represent changes between amino acids with different charges. Conservation plot scores reflect the nature of the change in specific sites: high scores are associated with changes with similar biological properties. Alignment was produced using the software Jalview version 2.11.1.7. (**B**) Phylogenetic analysis conducted by maximum likelihood method and the Kimura-2 parameter model showing the diversity of *E165R* gene of ASFV in the field. Based on the cluster, distribution isolates were categorized in four main groups. Numbers above internal branches represent bootstrap values (1000 repetitions). (**C**) The graphic represents the ratio dN-dS at specific codon sites in the *E165R* gene of ASFV. Red asterisks represent codon sites evolving under purifying selection, and green asterisks represent codons evolving under positive selection. Analyses were conducted using the evolutionary algorithms FEL and MEME considering a cutoff value of *p* = 0.1.

In this context, the high conservation depicted for the *E165R* gene among representative ASFV strains (Figure 1A) was consistent with the previously described role in ASFV as dUTPase [12]. Interestingly, in the case of the pandemic Eurasian lineage, we found a 100% similarity of identity between the Georgia 2007/1 strain (representative of the pandemic lineage), and all different strains from this lineage reported from Europe or Asia. However, in case of the strains circulating in Africa associated with this lineage (MAL/19/Karonga), there is amino-acid change at residue 130 that changes the conserved amino acid aspartic acid (D) for asparagine (N). The same situation was seen for the strain Tanzania/Rukwa/2017/1, omitted in the analysis for the 100% of similarity identity with the strain MAL/19/Karonga.

Phylogenetically, it was possible to classify the *E165R* gene in four main genetic groups (I to IV). In the case of Georgia 2007/1 strain, we found the highest level of identity (~99.46%) with strains MAL/19/Karonga, Liv13/33 and 22653/Ca/2014. Conversely, the lowest levels of identity were predicted against strains associated with groups III and IV (~94.24) (Figure 1B).

Finally, to get more insights about the evolution of the *E165R* gene, we applied a systematic evolutionary analysis, as previously described [20]. No evidence of recombination was predicted during the evolution of the *E165R* gene, after representative ASFV sequences were evaluated by the genetic algorithm for recombination detection (GARD) [21]. Overall dN/dS ratio = 0.225, calculated by the single-likelihood ancestor counting algorithm (SLAC) [22], indicated that purifying selection is the main force shaping the evolution of the *E165R* gene. In this context, the comparison between synonymous (dS) (0.249) and non-synonymous (dN) (0.061) evolutionary rates at different codons of *E165R* showed that synonymous mutations are fixated four times faster than non-synonymous ones.

A total of 16 codons at positions (19, 33, 57, 63, 71, 73, 82, 85, 91, 94, 101, 107, 135, 137, 148, and 151) were detected by the algorithm fixed-effects likelihood (FEL) [22] evolving under purifying selection, indicating that evolution is preserving the integrity of these sites (Figure 1C). This assumption is consistent with the location of most of these sites in the trimer interface (polypeptide binding) and active sites of the dUTPase (between residues 31 and 120). Of these sites, residues 71, 73, 91, 94 and 101 represent highly conserved sites among DUTPase’s of different species [23], highlighting the relevance of the conservation of these sites during the evolution of *E165R* in ASFV. Despite the different amino-acid changes along the E165R protein, evidence of positive selection was only found at residue 8 by the mixed-effects model of evolution (MEME) algorithm [24]. The evolution of the rest of the codons evolving under rates of dN-dS >1 sites may be considered neutral (Figure 1C), so that amino-acid changes at these positions may not be giving an evolutionary advantage to E165R protein.

### 3.2. Development of the ASFV-G-ΔE165R Deletion Mutant

**Figure 2:**
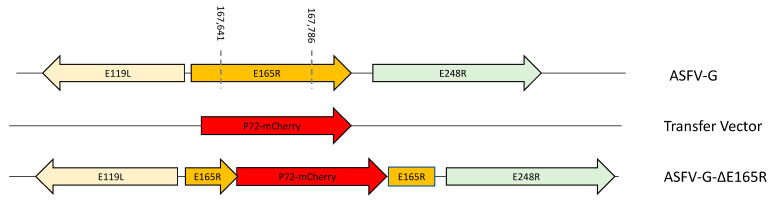
Schematic for the development of ASFV-G-∆E165R. The recombinant vector contains the p72 promoter and an mCherry cassette; the gene positions are indicated. The homologous arms were designed to have flanking ends to both sides of the deletion/insertion cassette. The nucleotide positions of the area that was deleted in the ASFV-G genome are indicated by the dashed lines. The resulting ASFV-G-∆E165R virus with the cassette inserted is shown on the bottom.

The high level of nucleotide and amino-acid conservation of the *E165R* gene among different ASFV isolates and along with its experimentally confirmed function as dUTPase [12,13,14,15] would indicate that E165R should play an important role in the process of virus replication. In fact, it was previously reported that an ASFV BA71V recombinant strain lacking *E165R* gene had a 90% decrease in their ability to replicate in primary swine macrophage cultures [12]. Therefore, to assess the role of the *E165R* gene during the process of virus replication in swine macrophage cultures and during the infection in domestic pigs, a recombinant virus having deleted the *E165R* gene was developed (ASFV-G-∆E165R) using the highly virulent ASFV Georgia 2007 isolate (ASFV-G) as the parental virus. In ASFV-G-∆E165R, the *E165R* gene was partially deleted and replaced with the p72mCherry∆E165R cassette by homologous recombination [4]. An area covering 146-bp (between nucleotide positions 167,641 and 167,786) was deleted from the ASFV-G genome, substituting the *E165R* gene with a 1226-bp cassette containing the p72mCherry construct. The N-terminus 173 -bp of *E165R* and the C-terminus 179bp were left intact so as not to disrupt the promoters of E119L or E248R (see Section 2) (Figure 2). Therefore, as designed, the reminding amino terminus of the *E165R* gene would not be expressed as truncated form of the protein as it lacks a viral promoter and open reading frame, the protein would be harboring only the first protein motif out of the functional 5 motifs originally described [12], particularly lacking the critical motif 3. It should be noted that this strategy closely resembles the design of the construct designed to delete the *E165R* gene in the BA71V virus [12].

The recombinant ASFV-G-∆E165R stock was purified after successive limiting dilution steps using primary swine macrophage cell cultures.

To assess the accuracy of the genomic modifications introduced into the ASFV-G-∆E165R genome, the full genome sequence was obtained by NGS using an Illumina NextSeq^®^ 500. A total of 963,119 reads were aligned to the ASFV genome and a comparative study between genomes of ASFV-G-∆E165R and ASFV-G demonstrated a 146 nucleotides deletion, and an insertion of 1226 nucleotides corresponding to the insertion of the p72-mCherry cassette sequence. No unwanted significant genomic changes were further identified in the ASFV-G-∆E165R genome. Additionally, NGS data demonstrated the absence of parental ASFV-G genome as a potential contaminant in the ASFV-G-∆E165R stock.

### 3.3. Replication of ASFV-G-∆E165R in Primary Swine Macrophages

As mentioned, a previous report demonstrated that a recombinant virus lacking the *E165R* gene showed a drastic decrease in its ability to replicate in primary swine macrophage cultures [12]. That virus was designed using the ASFV BA71V strain, a virus adapted to grow in Vero cells, as parental virus.

**Figure 3:**
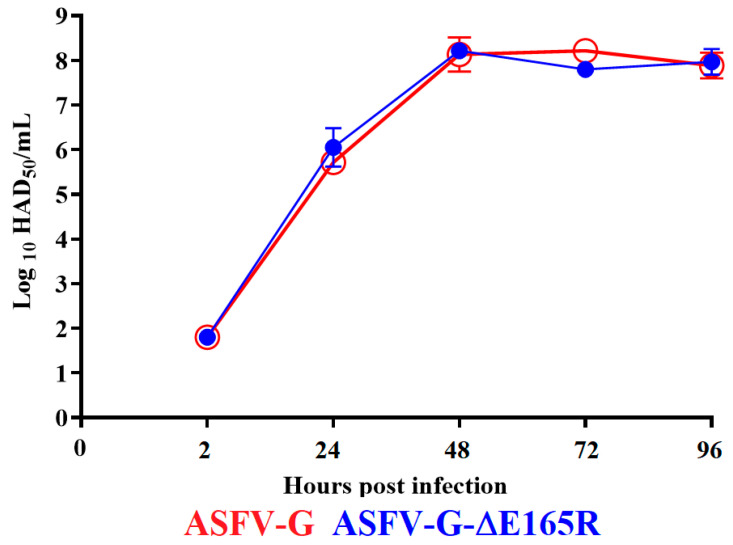
In vitro growth kinetics in primary swine macrophage cell cultures for ASFV-G-∆E1654R and parental ASFV-G (MOI = 0.01). Samples were taken from three independent experiments at the indicated time points and titrated in swine macrophages. Data represent means and standard deviations of three replicas. Sensitivity using this methodology for detecting virus is ≥log10 1.8 HAD_50_/mL.

To assess the effect of the deletion of the *E165R* gene during the process of virus replication of the highly virulent ASFV-G, the ability of ASFV-G-∆E165R to replicate in swine macrophages was evaluated. A multistep growth curve in primary swine macrophage cultures was performed to compare the replication kinetics between ASFV-G-∆E165R and the parental ASFV-G. Macrophage cultures were infected at a MOI of 0.01 with either ASFV-G-∆E165R or ASFV-G and virus titers were assessed at 2, 24, 48, 72, and 96 h post-infection (pi). Results demonstrated that ASFV-G-∆E165R showed a kinetics of replication indistinguishable from that of the parental ASFV-G (Figure 3), indicating that deletion of *E165R* gene does not affect the ability of replication of the ASFV in swine macrophages.

This result apparently contradicts the previous results reported using the ASFV BA71V strain [12]. It is possible that differences could be ascribed to the inherent characteristics of the BA71V strain. ASFV BA71V has been adapted to grow in a monkey-derived cell line, Vero cells, and the process of adaptation has caused a large deletion on both the left and right variable region of virus genome. The ASFV BA71V strain has lost around 11 genes that belong to the MGF360/505 [25], a relatively common process suffered by ASFV field isolates during the adaptation to grow in stabilized cell lines [17]. Conversely, ASFV-G-∆E165R parental virus, ASFV-G, is a field isolate that has always been replicated in primary swine macrophage cultures; therefore, it did not suffer any detectable significant genomic alteration. Therefore, the phenotype ASFV-G-∆E165R is strictly ascribable to the deletion of the *E165R* gene and not to other genomic changes potentially involved in the process of virus replication. It is possible that additional genomic modifications already present in the background of the ASFV BA71V would impede the potential substitution of the *E165R* gene function, as may happen in ASFV-G.

### 3.4. Assessment of ASFV-G-∆E165R Virulence in Swine

**Figure 4:**
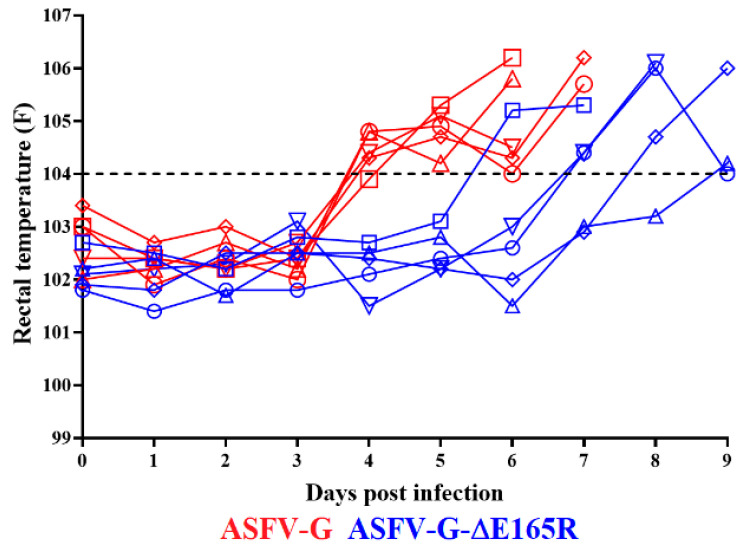
Evolution of body temperature in animals (5 animals/group) IM infected with 10^2^ HAD_50_ of either ASFV-G-∆E165R or parental ASFV-G. Unpaired *t* test with Welch correction using the Two-stage step-up (Benjamini, Krieger, and Yekutieli) method was conducted to assess statistical differences in temperatures between ASFV-G and ASFV-G-ΔE165R at different days post-infection. Significant differences between groups found at days four and five post-infection were evaluated using the false discovery rate method (FDR), being *p*-values < 0.05 considered significant.

**Figure 5:**
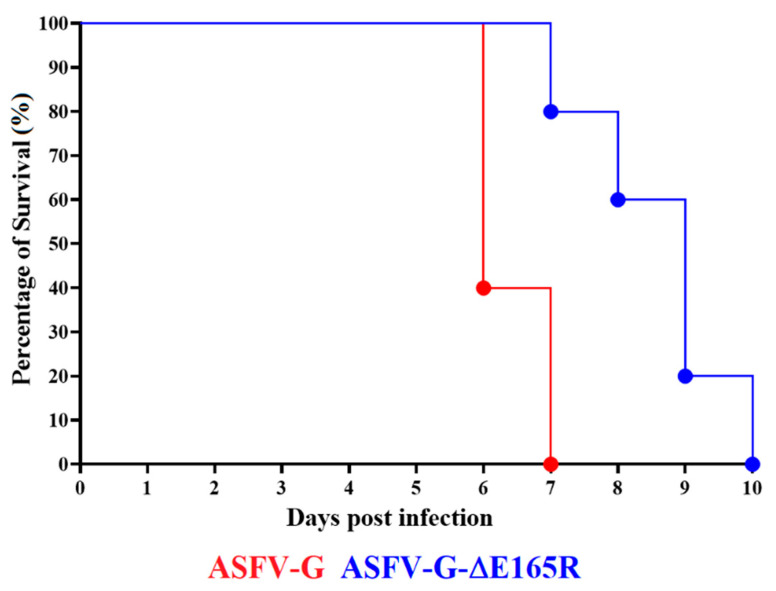
Evolution of mortality in animals (5 animals/group) IM infected with 10^2^ HAD_50_ of either ASFV-G-∆E165R or parental ASFV-G. Significant statistical differences were predicted between the two groups of pigs when evaluated by both the Log-rank (Mantel-Cox) test (*p*-value 0.008) and Gehan–Breslow–Wilcoxon test (*p*-value 0.009).

Once it was established that ASFV-G-∆E165R replicates in swine macrophage cultures as efficiently as the parental virus ASFV-G, it was important to evaluate the effect of deleting the *E165R* gene from the genome of ASFV-G in virulence in swine. A group of five domestic pigs was experimentally infected by IM inoculation of 10^2^ HAD_50_ of ASFV-G-∆E165R. The clinical evolution of that group was monitored for 28 days and compared with that of a control group, which was also IM inoculated but with 10^2^ HAD_50_ of ASFV-G. As expected, all animals inoculated with virulent ASFV-G presented an increase in body temperature (over 104 °F) on day 4 pi that rapidly progressed to a full clinical disease (depression, anorexia, staggering gait, diarrhea, and purple skin discoloration) (Figure 4 and Figure 5) with all animals euthanized by day 6–7 pi.

Animals inoculated with ASFV-G-ΔE165R also presented a lethal clinical form of the disease, though with a small delay. One of the animals started showing high body temperature by day 6 pi, followed by another two on day 7, and the last two animals in days 8 and 9 pi, respectively. All the animals clinically worsened in the following days, being euthanized between day 7 and 10 pi. Significant statistical differences were detected in the patterns of body temperature and survival curves between the two groups of animals (Figure 4 and Figure 5). Nevertheless, besides the slight delay in the presentation of clinical signs of the disease and time of euthanasia in animals inoculated with ASFV-G-ΔE165R, deletion of *E165R* did not drastically influence in the virulence of ASFV-G in domestic pigs.

**Figure 6:**
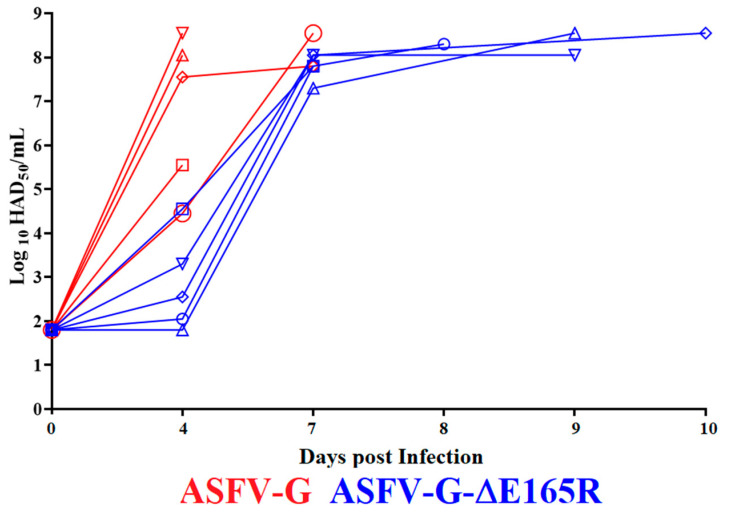
Viremia titers detected in pigs IM inoculated with 10^2^ HAD_50_ of either ASFV-G-∆E165R, or ASFV-G. Each curve represents individual animal titers. Sensitivity of virus detection: > log10^1.8^ TCID_50_/mL. Significant differences in viremia titers between the two groups were detected at day four post infection, calculated using the unpaired *t*-test considering the two-stage step-up (Benjamini, Krieger, and Yekutieli) method. The significance was evaluated using the false discovery rate method (FDR), being *p*-values < 0.05 considered significant. All calculations were conducted using the software Graphpad Prism version 9.2.0.

The replication in the animals of either ASFV-G-∆E165R or ASFV-G was evaluated by assessing viremia titers throughout the experimental period (Figure 6). As expected, viremias in animals inoculated with parental ASFV-G had high titers (ranging from 10^4.3^–10^8.55^ HAD_50_/mL) at day 4 pi, remaining high until the day all animals were euthanized. Viremias in animals inoculated with ASFV-G-∆E165R ranged from undetectable levels to 10^4.55^ HAD_50_/mL by day 4 pi. They reached high virus titers (between 10^7.13^–10^7.8^ HAD_50_/mL) by day 7 pi and remained high until all animals required euthanasia due to the severity of clinical disease. Therefore, viremia titers reflect the subtle differences in the clinical presentation of disease between animals inoculated with ASFV-G-∆E165R and parental ASFV-G.

Results reported herein indicate that deletion of *E165R* does not dramatically affect the ability of ASFV to replicate in macrophages, the natural target cell during infection in swine. These results clearly contradict previously reported results describing the *E165R* gene to be critical in virus replication in macrophages [12].

Differences between those results and these presented here may be due to the use of parental viruses with important genomic differences among them. While we used a natural highly virulent fieldisolate, those authors worked with a virus adapted to grow in Vero cells, with the concomitant loss of a large area on both the left and right sides of the virus genome, causing the deletion of approximately 11 viral genes [25]. Perhaps, the lack of several virus genes may account for the absence of a potential vicarious complementary function for the *E165R* gene, allowing the growth in swine macrophages as it happens in ASFV-G-∆E165R.

The results presented herein, showing that the deletion of the *E165R* gene does not affect virus replication in vitro or in vivo, as well as the virulence in domestic pigs, clearly indicate the inadequacy of any countermeasure therapeutic approach based in the inactivation or blockage of the *E165R* gene function, at least in the ASFV-G strain, as proposed by several reports [13,14,15].

In summary, we determined that *E165R* is a non-essential gene, since its deletion from the ASFV-G genome does not significantly alter virus replication in swine macrophage cultures and it is not involved in the process of virus virulence in domestic animals.

## Data Availability

Not applicable.
